# Endoscopic ultrasound-guided fine-needle aspiration of pelvic lesions via the upper and lower gastrointestinal tract approaches

**DOI:** 10.1186/s12876-020-01582-8

**Published:** 2021-01-06

**Authors:** Naoki Mita, Takuji Iwashita, Akihiko Senju, Hironao Ichikawa, Yuhei Iwasa, Shinya Uemura, Ichiro Yasuda, Masahito Shimizu

**Affiliations:** 1 First Department of Internal Medicine, Gifu University Hospital, 1-1 Yanagido, Gifu, 501-1194 Japan; 2 Third Department of Internal Medicine, University of Toyama Hospital, Toyama, Japan

**Keywords:** Pelvic lesion, Malignant lymphoma, Fine needle biopsy

## Abstract

**Background:**

Combining upper and lower gastrointestinal tract (GI) approaches allows expansion of endoscopic ultrasound-guided fine-needle aspiration (EUS-FNA) indications for pelvic lesions. The upper GI approach has been used for pelvic lesions around the level of the aortoiliac bifurcation in our institution. The aim of this study is to evaluate the feasibility and safety of EUS-FNA for pelvic lesions via the upper and lower GI approaches.

**Methods:**

All consecutive patients who underwent EUS-FNA for the pelvic lesion between January 2008 and December 2018 were retrospectively analyzed. Pelvic lesions were defined as lesions located around and below the aortoiliac bifurcation level. The primary outcome was technical success rate, and the secondary outcomes were the diagnostic capability of EUS-FNA for malignancy and the safety.

**Results:**

EUS-FNA for pelvic lesions was performed in 49 patients: upper and lower GI approaches were used in 28 and 21 patients, respectively. The technical success rates were 91.8% (45/49) in all patients: 89.3% (25/28) and 95.2% (20/21) with the upper and lower GI approaches, respectively. Among patients who achieved technical success, the diagnostic accuracy for malignancy was 97.8% (44/45) in all patients: 100% (25/25) and 95.0% (19/20) with the upper and lower GI approaches, respectively. One (2.0%) patient developed an adverse event of sigmoid colon perforation.

**Conclusions:**

EUS-FNA for pelvic lesions via the upper and lower GI approaches was a safe, feasible, and effective method, although careful endoscopic manipulation is required to avoid perforation, especially with the lower GI approach. Further large-scale, well-designed studies are needed to validate our findings.

## Background

Endoscopic ultrasound-guided fine-needle aspiration (EUS-FNA) has been a standard technique to obtain pathological specimens from lesions around the upper gastrointestinal tract (GI)[1], such as pancreatic tumors [2, 3], upper abdominal lymph nodes [4, 5], mediastinal masses [6], or upper gastrointestinal submucosal tumors [7, 8]. However, the feasibility and safety of EUS-FNA for pelvic lesions have not been well studied, although EUS-FNA for pelvic lesions via the lower GI approach was reported in several studies, including one systematic review and meta-analysis [9–11], in which target lesions were limited around the rectal or perirectal area. As our previously reported case series [12], the lesions around the level of the aortoiliac and internal–external iliac bifurcation could be approached with the upper GI, whereas other pelvic lesions located below that level could be approached with the lower GI. In our institution, the initial approach method during EUS-FNA, either the upper or lower GI, has been decided based on the above criteria.

Therefore, we conducted this retrospective study to evaluate the feasibility, efficacy, and safety of EUS-FNA for pelvic lesions via the upper or lower GI approach and the reference method to decide the approach route, either the upper or lower GI.

## Methods

### Study design and patient selection

This was a retrospective study conducted at a single academic care center (Gifu University Hospital). The database analysis including all EUS-FNA procedures between January 2008 and December 2018 was performed to identify patients who underwent EUS-FNA for pelvic lesions. Pelvic lesions were defined as lesions located in the pelvic area around and below the aortoiliac bifurcation level on computed tomography (CT). This study had no exclusion criteria. All patients provided written informed consent for EUS-FNA. The study was conducted in accordance with the human and ethical principles of research set forth by the Helsinki guidelines. The study protocol was approved by the Institutional Review Board of Gifu University Hospital.

### Selection of approach route and EUS-FNA techniques

The location of lesions was evaluated by CT before EUS-FNA. The approach route via either the upper or lower GI for EUS-FNA was decided based on the location of the lesion on CT. The selection reference was as follows: the upper GI approach for pelvic lesions located around the aortoiliac and internal–external iliac bifurcation levels and the lower GI approach for those around the rectum and sigmoid colon. All endoscopic ultrasound-guided (EUS) procedures were performed using an electronic linear scanning video echoendoscope with an oblique forward optical view (GF-UC240P-AL5 or GF-UCT260; Olympus, Tokyo, Japan).

In the upper GI approach, EUS scanning for lesions was first attempted from the stomach followed by the second portion of the duodenum (D2) with the pulling position as that used in endoscopic retrograde cholangiopancreatography. In the EUS scanning from D2, the aortoiliac bifurcation was visualized with further insertion of the EUS scope into the deep portion of D2 with keeping the longitudinal view of the aorta. In patients with lesions that can be visualized from both the stomach and D2, fine-needle aspiration (FNA) was performed from either approach route per the operator’s decision for a more reliable and safe EUS-FNA. In the lower GI approach, patients underwent screening colonoscopy to evaluate possible underlying colonic diseases, such as advanced cancer, which could be an obstacle for EUS-FNA. The target lesion was visualized using the surrounding organs, such as the urinary bladder, prostate, or uterus, as the landmark, and EUS-FNA was performed from the rectum or sigmoid colon. Prophylactic antibiotics were administered at the operator’s discretion after performing EUS-FNA via the lower GI approach.

EUS-FNA was attempted using FNA or FNB needles. The used FNA needles were a 19-gauge needle (Echotip, Cook, Winston-Salem, NC, USA; Expect, Boston Scientific, USA; EZ shot 3 plus, Olympus, Tokyo, Japan), 22-gauge needle (SonoTip Pro Control, Medi-Globe GmbH, Germany), or 25-gauge needle (Echotip; Cook, Winston-Salem, NC, USA). The FNB needles were a 19-gauge needle (Acquire, Boston Scientific) or 22-gauge needle (Acquire, Boston Scientific). EUS-FNA was performed mainly using a 10 or 20 cc negative pressure or a slow-pull technique to apply a minimal negative pressure. The obtained specimen was macroscopically evaluated, and whitish or yellowish pieces of tissue were fixed in formalin for histological evaluation, as we previously reported [13]. Then, a smear was made on the remaining specimen for cytologic evaluation and fixed in absolute alcohol. Rapid on-site cytologic evaluation was unavailable in our hospital. EUS-FNA was performed on an outpatient basis. After the FNA, patients were monitored for immediate adverse events (AEs) in the recovery room at least for 2 h. Additional examinations, such as blood tests or CT, were performed as necessary. The AEs were evaluated according to the American Society for Gastrointestinal Endoscopy workshop report [14].

### Study outcomes, reference methods for the final diagnosis, and statistical analysis

The primary outcome was the technical success rate of EUS-FNA for pelvic lesions with our reference method to decide the approach route, either via the upper or lower GI. Secondary outcomes were diagnostic capability of EUS-FNA and safety. The final diagnosis was obtained using the following references: (1) surgical diagnosis based on the resected specimen, including autopsy findings; (2) positive FNA diagnosis for malignancy with a compatible clinical course; (3) negative FNA diagnosis for malignancy with a lack of deterioration or spontaneous resolution with a minimal clinical follow-up time of 6 months; or (4) diagnosis for benign disease based on the imaging findings with absence of progression with a minimal clinical follow-up time of 12 months; (5) diagnosis for malignant disease based on the specimen which obtained by the method other than EUS-FNA or surgery. The technical success was defined as the successful completion of EUS-FNA for the targeted lesion. Continuous variables are presented as median and range. The outcome parameters were calculated with 95% confidence interval (95% CI). All statistical analyses were performed using JMP software, version 14.0.0 (SAS Institute Inc., Cary, NC, USA).

## Results

### Patient and lesion characteristics

Forty-nine patients underwent EUS-FNA for pelvic lesions at our institution from January 2008 to December 2018; 28 via the upper GI approach and 21 via the lower GI approach. Basic characteristics of patients are shown in Table 1.

**Table 1:** Patient and lesion characteristics

	Overall	Upper GI	Lower GI
Number of patients, n	49	28	21
Age, y.o., median (range)	69 (32–87)	68 (32–87)	72 (48–83)
Gender, male/female. n	28/21	12/16	16/5
Size of lesions, mm, median (range)	40 (13–135)	26 (32–87)	58 (13–135)
Target lesions, n (%)			
Pelvic LN	33 (67.3)	23 (82.1)	10 (47.6)
Pelvic mass	7 (14.3)	2 (7.1)	5 (23.8)
Retroperitoneal mass	3 (6.1)	3 (10.7)	–
Submucosal tumor	6 (12.2)	–	6 (28.6)

LN, lymph node; GI, gastrointestinal tract

### Final diagnosis

The final diagnoses in the upper GI group were malignancy in 24 patients (85.7%)—malignant lymphoma in 19, lymph node metastasis of malignant tumor in 4 (ovarian cancer in 2, gallbladder cancer in 1 renal cancer in 1), and carcinoma of unknown primary cancer in 1. In the remaining 4 patients (14.3%), the final diagnoses were benign—retroperitoneal fibrosis in 3 and lipoma in 1. The final diagnoses in the lower GI group were malignancy in 18 patients (85.7%)—malignant lymphoma in 6, lymph node metastasis of malignant tumor in 5 (bladder cancer recurrence after surgery in 2, rectal cancer recurrence after endoscopic treatment in 2, gallbladder cancer in 1), gastrointestinal stromal tumor in 4, carcinoma of unknown primary in 1, peritoneal mesothelioma in 1, and rectal invasion of bladder cancer in 1. In the remaining 3 patients (14.3%), the final diagnoses were benign—schwannoma in 1, Castleman disease in 1, and nonspecific lymphadenopathy in 1. The final diagnoses were obtained based on the surgical pathology in 12 patients, EUS-FNA results with the clinical course and median follow-up period of 27.2 (range, 6.3–103.3) months in 35 patients, the result of mucosal biopsy of the small intestinal lesion during double-balloon enteroscopy in 1 patient, and the imaging findings with the clinical course and follow up period of 41.3 months in 1 patient (Table 2).

**Table 2:** Final diagnoses of the patients with pelvic lesions who underwent EUS-FNA

	Overall n = 49	Upper GI n = 28	Lower GI n = 21
Malignant diseases, n (%)	42 (85.7)	24 (85.7)	18 (85.7)
Malignant lymphoma	25 (51.0)	19 (67.9)	6 (28.6)
LN metastasis of malignant tumor	9 (18.4)	4 (14.3)	5 (23.8)
Carcinoma of unknown primary	2 (4.1)	1 (3.6)	1 (4.8)
Gastrointestinal stromal tumor	4 (8.2)	–	4 (19.0)
Peritoneal mesothelioma	1 (2.0)	–	1 (4.8)
Rectal invasion of bladder cancer	1 (2.0)	–	1 (4.8)
Benign diseases, n (%)	7 (14.3)	4 (14.3)	3 (14.3)
Retroperitoneal fibrosis	3 (61.2)	3 (10.7)	–
Lipoma	1 (2.0)	1 (3.6)	–
Schwannoma	1 (2.0)	–	1 (4.8)
Castleman disease	1 (2.0)	–	1 (4.8)
Non-specific lymphadenopathy	1 (2.0)	–	1 (4.8)

EUS-FNA, endoscopic ultrasound-guided fine needle aspiration; LN, lymph node; GI, gastrointestinal tract

### Technical performance

**Fig. 1:**
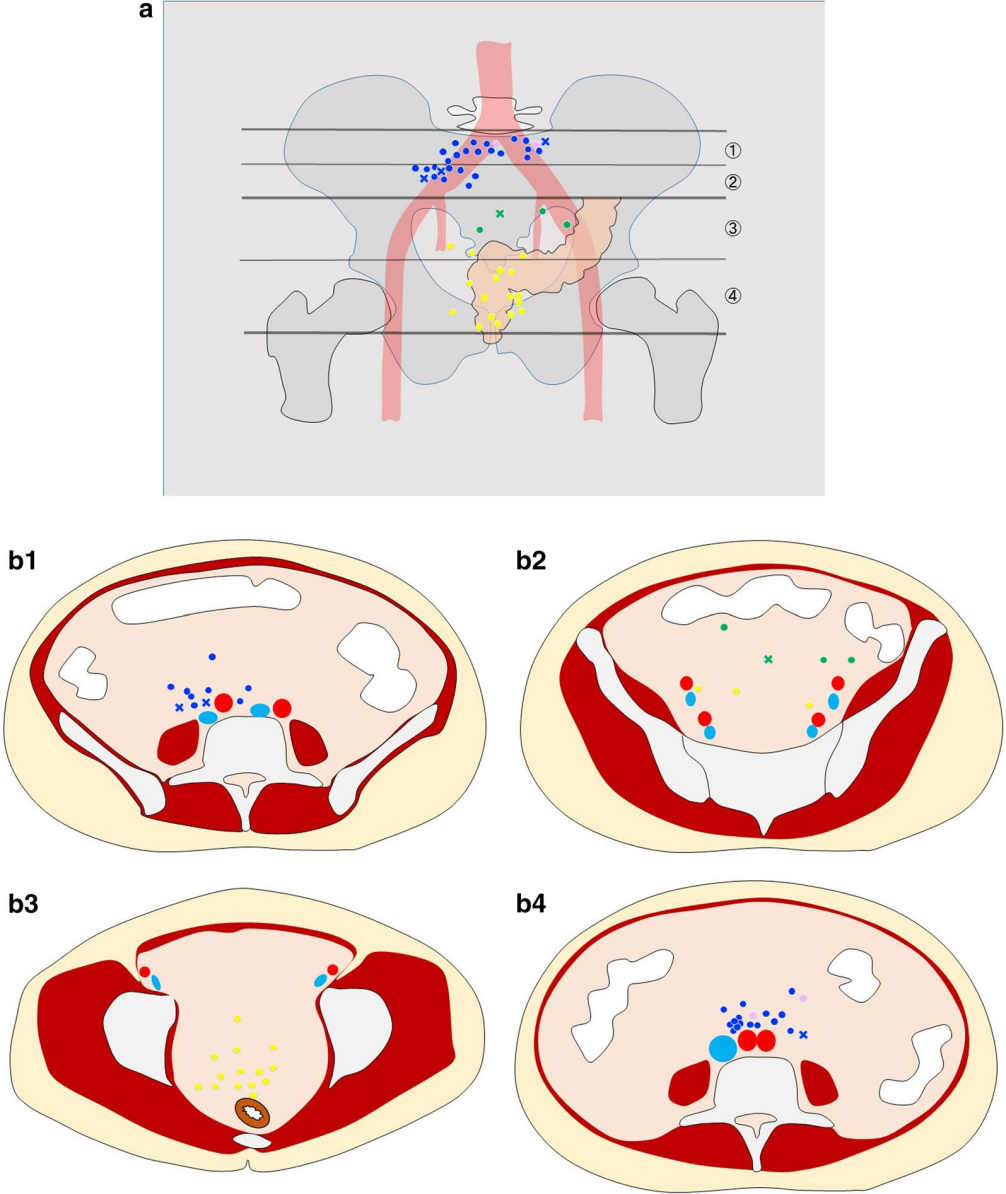
The location of the lesions, access route, and feasibility of endoscopic ultrasound-guided fine-needle aspiration. **A** The pelvic cavity was divided into four levels—the aortoiliac bifurcation, internal–external iliac artery bifurcation, sigmoid colon, and rectum. Circle mark indicates successful puncture, although cross mark is unsuccessful puncture. Color indicates puncture sight as following: blue, the second portion of the duodenum; pink, stomach; green, sigmoid colon; yellow, rectum. **B1** Axial image of the aorto-iliac bifurcation level. **B2** Axial image of the internal–external iliac artery bifurcation level. **B3** Axial image of the sigmoid colon level. **B4** Axial image of the rectum level

The technical success rates were 91.8% (45/49) in overall patients—89.3% (25/28) and 95.2% (20/21) with the upper and lower GI approaches, respectively. The FNA was unsuccessful via the upper GI approach because of the existence of vessels in the puncture route in 2 patients (1 in the aorta and 1 in the inferior vena cava), and the lesion was not detected in 1 patient via the upper GI approach. In the lower GI approach, FNA failed in 1 patient in whom the procedure was complicated by sigmoid colon perforation during the scope insertion. Emergent surgery was required in this patient. Among these 4 patients in whom FNA was unsuccessful, the final diagnoses were obtained based on the surgical pathology in 3 patients (renal cell carcinoma metastasis in 1, retroperitoneal fibrosis 1, and nonspecific lymphadenopathy in 1). In the remaining 1 patient, further imaging studies including CT, magnetic resonance imaging (MRI), and positron emission tomography CT (PET-CT) indicated suspicious of lipoma, and follow-up imaging studies showed no progression for 41.4 months. Regarding the type of needles, we used a 19-gauge FNA needle in 36 cases, 22-gauge FNA needle in 2 case, 25-gauge FNA needle in 1 case, 19-gauge FNB needle in 1 case and 22-gauge FNB needle in 5 cases. No AEs were observed, except for one, sigmoid colon perforation. Therefore, the AE rate was 2.0% (1/49). The location of the target lesions for EUS-FNA and the feasibility of EUS-FNA are shown in Fig. 1 (Table 3).

**Table 3:** Procedure details and data related EUS-FNA

	Overall n = 49	Upper GI n = 28	Lower GI n = 21
Technical success rate of EUS-FNA, n (%)	45 (91.8)	25 (89.3)	20 (95.2)
Reasons for unsuccessful cases, n		Existence of vessels in the puncture route, 2 The lesion was not detected, 1	Sigmoid colon perforation, 1
Type of needle, n (%)			
19-gauge FNA needle	36 (73.5)	21 (75.0)	15 (71.4)
22-gauge FNA needle	2 (4.1)	1 (3.6)	1(4.8)
25-gauge FNA needle	1 (2.0)	1 (3.6)	–
19-gauge FNB needle	1 (2.0)	–	1 (4.8)
22-gauge FNB needle	5 (10.2)	2 (7.1)	3 (14.3)
Puncture site, n (%)		Duodenum, 23 (82.1) Stomach, 2 (7.1)	Rectum, 17 (81.0) Sigmoid colon, 3 (14.3)
The number of passes, n, median (IQR)	3 (2–3)	3 (2–3)	3 (2–3)
Adverse event, n (%)	1 (4.8)	–	Perforation, 1 (4.8)

EUS-FNA, endoscopic ultrasound-guided fine needle aspiration; GI, gastrointestinal tract; FNB, fine needle biopsy; IQR, interquartile range

### Diagnostic capability of EUS-FNA

Among patients who achieved technical success with EUS-FNA, the histological evaluation was feasible in 97.8% (44/45), although the cytological material was obtained in all patients. The overall sensitivity, specificity, positive predictive value, negative predictive value, and accuracy of EUS-FNA for malignancy were 97.6% (40/41; 95% CI 87.4–99.6%), 100% (4/4; 95% CI 51.0–100%), 100% (40/40; 95% CI 91.2–100%), 80.0% (4/5; 95% CI 37.6–96.4%), and 97.8% (44/45; 95% CI 88.4–99.6%), respectively (Table 4). The histological and cytological diagnostic capabilities for malignancy are shown in Tables 5 and Table 6. In 1 patient with false-negative FNA result, we performed double-balloon endoscopy because the wall thickening of the small intestine was detected by CT imaging. And then, the final diagnosis of malignant lymphoma was obtained with mucosal biopsy of the small intestinal lesion.

**Table 4:** The overall diagnostic capability for malignancy in EUS-FNA for pelvic lesions

	All patients % (n, 95%CI)	Upper GI % (n, 95%CI)	Lower GI % (n, 95%CI)
Sensitivity	97.6 (40/41, 87.4–99.6)	100 (23/23, 85.7–100)	94.4 (17/18, 74.2–99.0)
Specificity	100 (4/4, 51.0–100)	100 (2/2, 34.2–100)	100 (2/2, 34.2–100)
PPV	100 (40/40, 91.2–100)	100 (23/23, 85.7–100)	100 (17/17, 81.2–100)
NPV	80.0 (4/5, 37.6–96.4)	100 (2/2, 34.2–100)	66.7 (2/3, 20.8–93.9)
Accuracy	97.8 (44/45, 88.4–99.6)	100 (25/25, 86.7–100)	95.0 (19/20, 76.4–99.1)

EUS-FNA, endoscopic ultrasound-guided fine needle aspiration; GI, gastrointestinal tract; CI confidence interval; PPV, positive predictive value; NPV, negative predictive value

**Table5:** The histological diagnostic capability for malignancy in EUS-FNA for pelvic lesions

	All patients % (n, 95%CI)	Upper GI % (n, 95%CI)	Lower GI % (n, 95%CI)
Sensitivity	97.5(39/40, 87.1–99.6)	100 (23/23, 85.7–100)	94.1 (16/17, 73.0–90.0)
Specificity	100 (4/4, 51.0–100)	100 (2/2, 34.2–100)	100 (2/2, 34.2–100)
PPV	100 (39/39, 91.0–100)	100 (23/23, 85.7–100)	100 (16/16, 80.6–100)
NPV	80.0 (4/5, 37.6–96.4)	100 (2/2, 34.2–100)	66.7 (2/3, 20.8–93.9)
Accuracy	97.7 (43/44, 88.2–99.6)	100 (25/25, 86.7–100)	94.7 (18/19, 75.4–99.1)

EUS-FNA, endoscopic ultrasound-guided fine needle aspiration; GI, gastrointestinal tract; CI confidence interval; PPV, positive predictive value; NPV, negative predictive value

**Table6:** The cytological diagnostic capability for malignancy in EUS-FNA for pelvic lesions

	All patients % (n, 95%CI)	Upper GI % (n, 95%CI)	Lower GI % (n, 95%CI)
Sensitivity	82.9 (34/41, 68.7–91.5)	78.3 (18/23, 58.1–90.3)	88.9 (16/18, 67.2–96.9)
Specificity	75.0 (3/4, 30.1–95.4)	100 (2/2, 34.2–100)	50.0 (1/2, 9.5–90.5)
PPV	97.1 (34/35, 85.5–99.5)	100 (18/18, 82.4–100)	94.1 (16/17, 73.0–99.0)
NPV	30.0 (3/10, 10.8–60.3)	28.6 (2/7, 8.2–64.1)	33.3 (1/3, 6.1–79.2)
Accuracy	82.2 (37/45, 68.7–90.7)	80.0 (20/25, 60.9–91.1)	85.0 (17/20, 64.0–94.8)

EUS-FNA, endoscopic ultrasound-guided fine needle aspiration; GI, gastrointestinal tract; CI confidence interval; PPV, positive predictive value; NPV, negative predictive value

## Discussion

The reference method to decide an approach route, either with the upper GI or lower GI, during EUS-FNA for pelvic lesions was evaluated as the technical success in this study. The upper GI approach was selected for pelvic lesions located around the aortoiliac and internal–external iliac bifurcation levels and the lower GI approach was chosen for those located below the level of the internal–external iliac bifurcation. The technical success rates in this study were considered as relatively high and were 91.8% (45/49) in overall—89.3% (25/28) with the upper GI approach and 95.2% (20/21) with the lower GI approaches. The EUS-FNA failed in 2 of 4 patients because of interposing large vessels on the puncture line. Unnecessary procedures can be avoided if large vessel disruption during EUS-FNA can be estimated based on CT findings; however, the estimation could be difficult because the intestines are movable during the insertion of EUS, which change the positional relation between the lesion and the scope. Considering the high technical success rate with our approach method and the difficulty in estimating the interposing vessels during FNA, our determination method of the approach route in EUS-FNA for pelvic lesions can be considered appropriate.

The accuracy of EUS-FNA has been reported as 96% in pancreatic lesions [2, 15], 87.5%–98% in upper abdominal lymph nodes [4, 16], and 70%–90% in upper gastrointestinal submucosal lesions [17] in previous studies. Among patients who achieved a technical success with EUS-FNA in this study, the overall accuracy for malignancy with EUS-FNA for pelvic lesions was 97.8% (44/45), which was comparable to the these reported diagnostic capability. In addition, the 19-gauge needle was most frequently used for FNA in this study, which might contribute to the high histological specimen acquisition rate (97.8%) and high diagnostic capability since almost half of the final diagnosis in our present study was malignant lymphoma which requires a histological analysis including immune histochemical staining [18, 19]. Large-bore needles generally compromise maneuverability because of its rigidity and stiffness, making the puncture difficult during EUS-FNA, especially via the transduodenal approach. Our high feasibility might be not applicable for other centers, considering our extensive experience with EUS-FNA using a 19-gauge needle. Actually, Attili et al. reported that the histological sample acquisition rate and overall diagnostic accuracy of transduodenal EUS-FNB using 19-gauge needle as only 76.8% and 73.6%, respectively[20]. Recently, new fine-needle biopsy (FNB) needles have been developed; they have been reported to have excellent tissue acquisition and histological diagnostic rates, even with a smaller gauge needle size[21–24]. Therefore, the size and type of FNA needle should be chosen considering the location and shape of the EUS scope, lesion size, and operator’s experience.

Regarding the safety of EUS-FNA for the pelvic lesion via the upper GI, although no AEs were recognized in this study, the echoendoscope has to be pushed downward against the duodenal or gastric wall to visualize the pelvic lesion, which might increase the risk of perforation or bleeding. The operator should be cautious of not using too much pushing force to visualize the lesions. In a systematic review and meta-analysis of EUS-FNA via the lower GI approach for pelvic lesions conducted by Han et al. [9], which included 10 studies with a total of 236 cases, AEs occurred in 1.7% (4/236) of cases and were 2 cases of abscesses after EUS-FNA for cystic lesions, 1 case of gross hematuria and 1 case of hemorrhage. They concluded EUS-FNA via the lower GI for pelvic lesions is a safe procedure with low AE rate, although we think that indication of EUS-FNA for cystic pelvic mass should be considered carefully. In our study, only 1 patient developed an AE of sigmoid colon perforation during the EUS-FNA via the lower GI. Considering the limited angulation of a convex-type EUS with oblique viewing, deep insertion of the EUS scope into the sigmoid colon is challenging and requires careful maneuver of the scope to minimize the risk of preformation. Recently, forward-viewing EUS (FV-EUS) having forward optical view and wider scope angulation has been developed; they can be theoretically used as a regular colonoscope[25]. In a study by Thinrungroj et al., EUS-FNA using FV-EUS and fluoroscopy via the lower GI approach, including the deep colon approach, was successfully performed in 13 patients without any AEs [26]. The authors concluded that FV-EUS under fluoroscopy guidance might be an easy, safe, and effective technique for transcolonic EUS-FNA. FV-EUS for pelvic lesions can expand the indication of EUS-FNA for pelvic lesions via the lower GI approach and improve the safety especially during the scope insertion.

This study has several limitations. A retrospective study design in a single center with a small sample size might cause biases in the patient selection and external validity of the procedure. A retrospective study design also did not allow to include all patients who had pelvic lesions on imaging studies. The wide period of inclusion and the use both of FNA and FNB needle might cause inconsistency of procedures. The final diagnoses were determined according to both surgical and FNA results, which might cause misdiagnosis in indolent tumors, even with a minimal follow-up period of > 6 months.

## Conclusions

EUS-FNA for pelvic lesions via the upper and lower GI approaches is a feasible, safe, and reliable procedure, and the internal–external iliac artery bifurcation level can be a good indicator to decide the approach route, either via the upper or lower GI. Larger scale multicenter studies are required to validate the feasibility and safety of EUS-FNA for pelvic lesions and our approach for pelvic lesions with respect to selection of the upper or lower GI approaches.

## Data Availability

All data generated or analyzed during this study are included in the tables. The study data is available from the corresponding author upon reasonable request.

## Abbreviations

EUS-FNA: Endoscopic ultrasound-guided fine needle aspiration
GI: Gastrointestinal tract
D2: 2nd portion of the duodenum
CI: Confidence interval
MRI: Magnetic resonance imaging
PET-CT: Positron emission tomography-CT
FV-EUS: Forward-viewing therapeutic linear echoendoscope
